# Defining the Black population in Canadian health research: a scoping review

**DOI:** 10.1186/s12889-026-27940-x

**Published:** 2026-06-03

**Authors:** Marie-Claire Uwamahoro, James Idowu, Nashit Chowdhury, Sumedh Bele, Naomi Popeski, Francis Boakye, Charles Odame-Ankrah, Régine Uwibereyeho King, Bukola Salami, Doreen M. Rabi, Tanvir C. Turin

**Affiliations:** 1 Department of Community Health Sciences, Cumming School of Medicine, University of Calgary, Calgary, AB Canada; 2 Nuffield Department of Orthopaedics, Rheumatology and Musculoskeletal Sciences, University of Oxford, Oxford, UK; 3 Action Dignity Society, Calgary, AB Canada; 4 Calgary African Community Collective, Calgary, AB Canada; 5 Faculty of Social Work, University of Calgary, Calgary, AB Canada; 6 Faculty of Nursing, University of Calgary, Calgary, AB Canada; 7 Department of Medicine, Cumming School of Medicine, University of Calgary, Calgary, AB Canada; 8 Department of Family Medicine, Cumming School of Medicine, University of Calgary, Calgary, AB Canada

**Keywords:** Black, African, Caribbean, Canada, Definition, Health research

## Abstract

**Background:**

Inconsistent definitions, labels, and classifications of the Black population in Canadian health research raises an important question: Who is considered Black? This inconsistency can unintentionally hinder the comparability and effectiveness of studies, potentially leading to misinformed healthcare policies, ineffective interventions, and perpetuated health disparities. The purpose of this scoping review was to explore how the Black population is defined and represented in Canadian health research.

**Methods:**

A scoping review was conducted following the methodology of Arksey and O’Malley and the methodological advancements made by Levac and colleagues. Databases, including MEDLINE (Ovid), PsycINFO, EMBASE, PubMed, CINAHL, Scopus, ProQuest, OAISter and Google, were searched from their inception until November 2023. Two reviewers independently screened titles, abstracts and full texts for inclusion, with discrepancies resolved through third-party adjudication. Studies were included if they were conducted in Canada, reported race or ethnicity-specific outcomes, and focused on health. Data were extracted using a customized form developed in Covidence software. Study characteristics were summarized in SPSS 29, and NVIVO 14 was used for reflective thematic analysis to identify terminologies and definitions.

**Results:**

A total of 11,935 records were retrieved, and 517 were included. Health researchers in Canada used various terms to describe Black participants, with “Black” being the most frequent, alongside terms like “Caribbean” and “African.” Historical terms such as “Negro” also appeared in older studies. About 53% of the studies lacked descriptions of the terms used. Only a limited number of studies explicitly defined the terms they used, connecting their definitions of the Black population to historical events like slavery or migration from Africa to Canada. However, definitions of similar terms appear to be different across studies.

**Conclusion:**

The findings highlight a significant inconsistency and lack of standardization in the terminology and definitions of Black populations in health research. This inconsistency not only undermines research validity but also poses a substantial barrier to developing effective health policies and interventions tailored for Black communities. The results underscore the urgent need for standardized guidelines and community-informed and culturally responsive frameworks in health research to ensure inclusivity, precision, and equity in studying Black populations.

**Supplementary Information:**

The online version contains supplementary material available at 10.1186/s12889-026-27940-x.

## Background

Canada’s Black population is a dynamic and expanding demographic, encompassing individuals with ancestral ties to Africa, the Caribbean, and various global regions [1]. This diversity is reflected in a range of cultural traditions, languages, and migration histories, including the legacy of slavery [2]. As a result, the Black population in Canada occupies a unique position at the intersection of multiple identities and experiences. The formation of community among Black Canadians often varies, shaped by `factors such as country of origin, tribe, and other shared interests [3]. Black communities also continue to face systemic inequities and racism, which affect their health outcomes, access to care, and representation in health research [4]. These systemic inequities substantially exacerbate existing health disparities, underscoring the urgent necessity for precise identification, accurate representation, and culturally informed approaches in research to ensure equitable and effective healthcare.

Health research is essential for developing evidence-based policies and interventions. Within Canada, the systematic collection of race-based data is increasingly recognized as essential for developing informed policies aimed at reducing health inequalities and promoting equity [5]. Nevertheless, a significant challenge remains the inconstant definitions, labels, and classifications for the Black population in Canadian health research [6]. Such inconsistency carries significant consequences, including misidentification of specific community health needs, incorrect data interpretation, and interventions that fail to address or can even exacerbate existing inequities. The terminology used to describe the Black population in Canada is extensive, and the interchangeable use of terms raises questions about who is truly considered Black [6]. Some terms include “Black individuals, people, or communities,” “Black Canadians,” “African Canadian Caribbean,” “Black Africans,” “African-Caribbean or African and Caribbean communities,” “Black Nova Scotians,” and “Black ethnicity “ [6].

Organizations such as the Canadian Institute for Health Information (CIHI) and Statistics Canada [7] have attempted to establish national standards for collecting race-based data in healthcare. However, these standards are not mandatory, and their implementation varies across Canada. This variability undermines the reliability, consistency, and practical applicability of health data, weakening the foundation upon which targeted interventions and effective policies depend. In addition, existing guidelines often conflate race with ethnicity and country of origin, further complicating clarity and consistency in research methodologies and interpretation. Researchers often develop their own definitions, the basis of which is not always clear. This lack of enforcement and clarity in approaches compromises the reliability of data, thus weakening research that informs critical health policy and intervention efforts.

Statistics Canada’s census methods define the Black population as comprising over 300 distinct ethnic or cultural origins and more than 450 mother tongues [1]. This immense diversity may tempt researchers to oversimplifying by using broad terms such as “Black” or taking shortcuts in terminology, risking the conflation of these varied subgroups. However, Black is an ascribed category that varies by location and is shaped by social, historical, and political contexts. For example, in Australia, the term “Black” is often associated with Indigenous populations [8]. In contrast, in South Africa, individuals of mixed ancestry would typically be classified as “Coloured“ [9] rather than Black. Such an approach fails to capture the unique experiences of specific communities, resulting in findings that lack relevance or applicability and undermining the utility of the research. Additionally, such oversimplification can erode trust in health research and further marginalize underrepresented groups, creating barriers to achieving true health equity. This scoping review aimed to systematically map the existing body of Canadian health research on Black populations, to identify how these populations are defined in Canada’s health research.

## Methods

The scoping review was conducted following the six-stage methodological framework initially described by Arksey and O’Malley [10] and subsequently refined by Levac et al. [11] The detailed methodology of this scoping review has been published elsewhere [12]. 

Our review sought to address two overarching research questions:

1. What terminologies and labels do researchers use to identify and categorize the Black population in Canadian health studies?
2. How do health researchers in Canada define the Black population?

The manuscript protocol had four questions [12], however we present two integrative questions here. During the analysis of the scoping review, we found that these four questions could be more clearly addressed through two broader questions: one focusing on the terminologies and labels used to identify and categorize Black populations, and the other focusing on how the Black population is defined in Canadian health research. This consolidation was also appropriate because many studies did not provide explicit definitions. Instead, definitions were often implied through the terminology used, the recruitment criteria, the population categories selected, or the explanations provided by the authors. Thus, the two revised questions allowed us to capture both explicit definitions and the broader processes through which meanings of “Black” were constructed in the literature.

We conducted a comprehensive search of MEDLINE, EMBASE, CINAHL and other social sciences, and education databases [12] from their inception to October 2023. We also performed a gray literature search using Google Scholar, ProQuest (theses and dissertations) and OAISter. Health sciences and other community organizations were also searched as described in our protocol [12]. The relevant keywords and synonyms related to the Black population such as ‘Black’, ‘African Canadian’ African- born’, ‘Afro- Caribbean’, and ‘Black minority’. The final list contained over 50 population search terms reflecting the diversity of Black communities in Canada [12]. Details of the databases and search strategies are detailed in the published protocol of this scoping review [12]. 

### Study selection

Studies were selected based on a prior determined inclusion and exclusion criteria. We included the studies if: studies included the Black population, conducted in Canada/include the Canadian population, studies focused on clinical, population and/or public health and reported race, ethnicity-specific outcomes or findings [12]. The details of the reviewers’ selection steps can be identified in the protocol [12]. 

### Data extraction

Two reviewers independently extracted data using a standardized data collection tool developed a priori. Data elements of interest included key characteristics of each study, such as the author(s), year of publication, title, aims, objectives, topics covered, study design and approach, setting, population size, and the focus on health. We extracted terminology related to “Black” populations from the studies, along with the explicit definitions of “Black” used by the authors when available. Definitions of the terminologies were tracked by analyzing how authors justified the use of terminologies and processes and mechanisms of recruitment. Initially, we examined the terminology used to describe the population in the study titles, objectives, or aims. Next, we investigated the sources of data. For example, we assessed whether the study utilized qualitative/quantitative methods such as participant interviews, surveys with self-administered questionnaires that included ethnicity self-identification, or other recruitment strategies (See Additional file 1).

### Data synthesis and reporting

The extent and nature of studies eligible for analysis are presented using descriptive statistics in tables and charts. We used IBM *SPSS* Statistics 29.0 to compute and analyze the characteristics of the studies such as the year of publication, study design, setting and sample, those that defined the term Black and those that engaged the community. The extracted texts such as the terminologies were imported and processed using NVivo 14 software. An inductive approach to data analysis was taken by conducting the reflective thematic analysis (RTA) of Braun and Clarke [13]. The explicit definitions were documented exactly as presented in the studies (Table 3).

### Patient and public involvement

This scoping review involved active participation from members of the Black communities, who contributed substantially to identifying key terms for developing culturally relevant and acute research strategies. They recommended terminologies that are closely associated with the Black population in Canada. This included terminology commonly utilized within their own communities as well as those terms used by the public to refer to the Black population. Additionally, community members critically co-reviewed and provided valuable feedback on the manuscript, ensuring cultural sensitivity, accuracy of representation, and relevance of findings to the communities discussed.

## Results

### Literature search overview

**Fig. 1:**
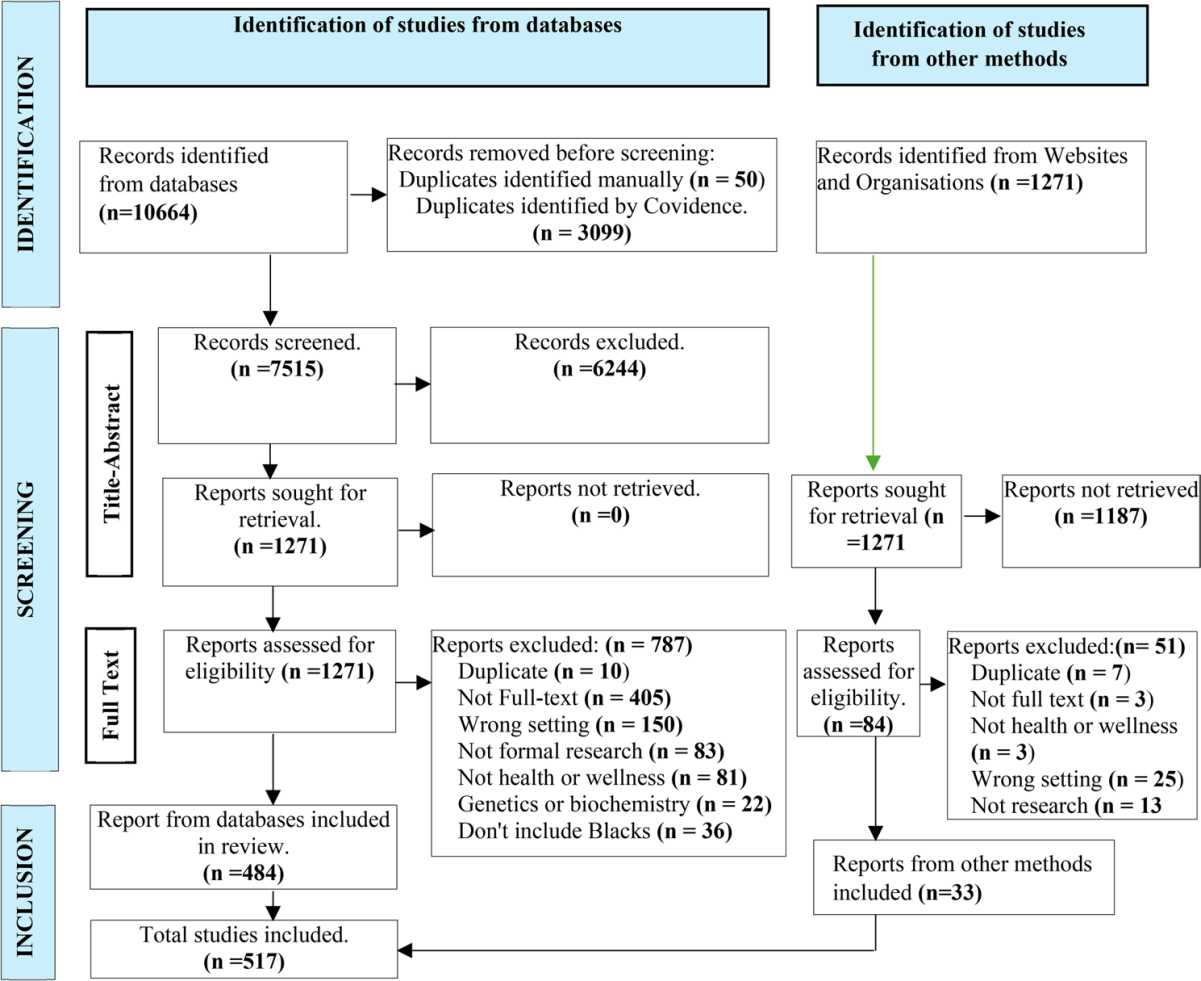
PRISMA flow diagram of the search and selection process for the scoping review [25]

The academic database search yielded 10,664 studies and an additional 1,271 studies were identified from the grey literature. After removing duplicates and applying the inclusion criteria 517 studies were included (484 from academic databases and 33 from the grey literature) (Fig. 1).

### Characteristics of the studies

**Fig. 2:**
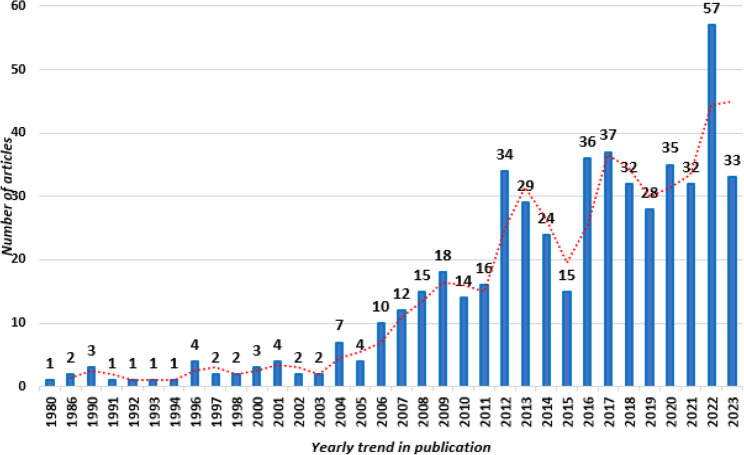
Publication frequency by year (1980–2023)

The quantitative studies (149 studies) were largely cross-sectional surveys (Table 1). Few were Randomized control and non-randomized experimental, 5 and 12, respectively. There were 119 qualitative studies. Clinical (*n* = 182) and community health (*n* = 158) areas accounted for over 65% of the identified studies. The most frequently studied topics were (1) HIV, (2) mental health, (3) diabetes, (4) cancer, (5) hypertension and cardiovascular disease and (6) COVID-19. The minimum of sample size was one person, and the maximum was 1,291,561. Ontario alone accounts for 57.4% of all studies included. Community engagement was infrequent with 19.9% of studies reporting to have engaged or consulted with community. In these studies, the level of engagement happened during the recruitment, where researchers reached out to communities or organizations that could facilitate the identification and enrolment of participants (Table 1). The number of studies that explicitly included Black populations has steadily increased since 1980 (see Fig. 2).

**Table 1:** Characteristics of the studies

Characteristics	Frequency (%)
Study designs (*n* = 517)	
Randomized control	5 (1)
Non-Randomized experimental	12 (2.3)
Cohort studies	72 (13.9)
Cross sectional	149 (28.8)
Case Control	6 (1.2)
Case series	1 (0.2)
Case report	3 (0.6)
Chart review	25 (4.8)
Other Quantitative	111 (21.5)
Qualitative	119 (23)
Government or Community	14 (2.7)
Focus of the Study (*n* = 517)	
Health Education	4 (0.8)
Clinical	182 (35.2)
Community Health	158 (30.6)
Health care system	35 (6.8)
Mental Health	84 (16.2)
Psychology	3 (0.6)
Wellness	42 (8.1)
Other	9 (1.7)
Province (*n* = 517)	
Alberta	45 (8.7)
British Columbia	21(4.1)
Manitoba	7 (1.4)
Nova Scotia	17 (3.3)
Quebec	64 (12.4)
Saskatchewan	1(0.2)
Northwest Territories	2 (0.4)
Two or more provinces	31 (6.0)
Entire Canada	31 (6.0)
Ontario	297 (57.4)
Newfoundland and Labrador	1 (0.2)
Definition of Black/terminologies (*n* = 517)	
Yes	245(47)
No	272(53)
Community engagement (*n* = 517)	
Yes	103 (19.9)
No	414 (80.1)
Level of engagement (*n* = 103)	
Proposal	9 (1.7)
Recruitment	88 (17)
Throughout the study	6 (1.2)
Sample of Black participants (*n* = 517)	
Minimum	1
Maximum	1,291,561

### Terminologies used to describe the Black population

We identified a range of terminologies associated with the Black population. Table 2 portrays the core terms while also listing their connected labels. Additionally, every connected label has an example of designated reference/s. The core terms are broader categories. For example, “Black“ [80],“African” [81, 82], “Caribbean” [18, 83], combination like “African and Caribbean” [66, 84], “BIPOC-Black” [46], “West Indian” [85], “Negroes” [45], “Nova Scotians” [86], as well as references to African regions [87, 88] (e.g., East, West, Horn of Africa) and subregions like Sub-Saharan [89]. Specific Africa or Caribbean countries names for example Ghana and Jamaica were also used [50]. 

**Table 2:** Terminologies used to describe the Black population

Core terms	Connected labels
African	“African,“ [26] “African (includes Afro-Caribbean),“ [27] “African American,“ [26–28]”African Canadian,“ [26, 28]”African Black,“ [29]
Caribbean	“Caribbean,“ [30] “Caribbeans black,“ [17] “Caribbean-Canadians (Black),“ [17] Caribbean: (specific country like Jamaica and Haiti) " “Central America/Caribbean,“ [28], “Non-Spanish Caribbean,“ [31] “South America Caribbean,“ [32] “Indo-Caribbean “ [33, 34]
Africa and Caribbean	“African-Caribbean [35]/Afro-Caribbean “ [29]”African and Caribbean“ [36]
African Caribbean Black (ACB)	“African, Caribbean and Black“ [37] “African, Caribbean, and other Black,“ [38],” “Caribbean and Black (ACB)“ [39] Multi-ethnic ACB“ [26]
Black	“Black [40]/black [40, 41],” “Black-African,“ [27] “Black American,“ [39] “Black Canadian,“ [26] “Black Canadian/American,“ [39] “Black Caribbean,“ [28] “Black Caribbean-Canadian,“ [39] “Black from Africa,“ [42] “Black from the Caribbean,“ [42] “Black Latin American,“ [39] “Black South or Central America,“ [28] Black settlers [43],“Black/Afro-Canadian,” [39]”Black/African + a country [44] (Barbarian, Haiti, Cuban)” [24, 29]
Negroe	Negroe [45]
BIPOC- Black,	BIPOC- Black, [46]
Visible minority	Visible Minority (Black) [46]
Africa subregions	“Central Africa,“ [30] “East Africa,” “Horn of Africa,“ [47] “Northeast African,” “Southern Africa sub-regions,” “Middle African“ [48] “West Africa“ [30]
Caribbean regions	Caribbean Region (e.g., Jamaican, Guyana, Trinidadian/Tobagonian) [48]
Specific name of the country of origin	Some examples="Jamaican-Ethiopia,” [49]”Grenadian Canadian,“ [49] “Bajan/Guyanese,“ [49] “Jamaican Canadian,“ [49] “Trinidadian,“ [49] “born in Ghana,“ [50] “Born in Jamaica,“ [50] “Ethiopia,“ [51] “Ghanaian,“ [52] “Haitians,“ [53] “Kenya,“ [51] “Nigerian,“ [54] “Republic of Democratic Congo,“ [55] “Rwanda,“ [52] “Somalia,“ [56] “Uganda“ [52]
West Indian	“West Indian“ [26]”"Black West Indian,“ [57] “Black West Indian (Caribbean) [58],” “Black West-Indian Canadian [59],” Caribbean/West Indian“ [26]
Nova Scotia	Indigenous Africa Nova Scotia [60] African Nova Scotian [60], Black Nova Scotians [61]
Associated variations based on terms like “origin”, “descendant”, “born”, “generation” “Primarily”	“African descent,“ [22] “African Origin,“ [62] “African, and Caribbean origins,” “black ancestry,“ [63]”Born in (country [50], region [64] or continent [65]), " “Caribbean descent,“ [66] “Caribbean origin,“ [27] “Continent of origin (Africa) [67]” “Country of origin within Africa,“ [68] “Native-born and foreign-born Black,” “Native-born Blacks,“ [69] “Nova Scotians of African descent,“ [62] “people of African descent,“ [2] “Sub-Saharan African origin,“ [70] “Sub-Saharan African-born,“ [71] “First- and second-generation Black“ [4] “Multigeneration African Canadians“ [22],” Afro-Caribbean“ [72] Primarily from Jamaica [73]…"Black primarily Caribbean” [74]
Descriptors (language, gender, individual, population, people, community, diaspora, immigrant, newcomer, ethnicity, race)	“Anglophone Afro-Caribbean “ [15] “French/English Caribbean [75], “Black/African communities [43]/diaspora,“ [22]”African Immigrant/refugees,“ [2] “Black individuals,“ [76] “African newcomers,“ [61] “Black people,“ [77] “People from Africa“ [43] “Black population,“ [78] “Multi-ethnic Black” [79] “Haitian ethnic origin“ [79]

In addition, various descriptors such as people (e.g., People of Black Africa) [90], origin (e.g., African origin) [91], descent (e.g., of African descent) [92], birthplace (e.g., Born in Ghana) [50], ethnicity (e.g., Black ethnicity) [93], race (e.g., Black race) [94], ancestry (e.g., Black ancestry) [63], primarily (e.g., Afro-Caribbean primarily from Jamaica) [73], and community (e.g., Black community) [86] were often used alongside these terminologies (see Table 2).

The retrieved articles did not make it possible to determine exactly when each term first began to appear in Canadian health research. However, some terminology patterns were observed. Terms such as “Negro” [45] did not appear in more recent articles, at least not in those published after 2000. By contrast, terms such as “Black” and “African” were present from the earliest articles included in this review through to more recent publications [39, 45]. Terms that are not currently included in CIHI standards, such as “BIPOC” [46] are still used in the literature and may include Black populations depending on the context. Similarly, terminology currently reflected in CIHI standards, such as “African, Caribbean and Black” (ACB) [41]appeared as early as 2003.

### Definitions of used terminologies

In certain instances, researchers did not provide a definition of the terminologies used. Over half of the reviewed studies (53%) failed to define their terminologies for the population involved, whether they referred to it as “Black” or used alternative terminologies displayed in Table 2. Among the 245 (47%) studies (Table 1), categorized as defining their terminologies only a small fraction—9, offered specific operational definitions (Table 3). In contrast, 238 studies took a more flexible approach by allowing participants to self-identify. In the few studies where researchers explicitly defined study populations, distinct approaches emerged (See Table 3).

**Table 3:** Operational definitions of used terminologies

Terminologies	Full definitions
Black Canadians [14]	“In this study, the operational definition of “Black Canadians” denotes people of African descent in Canada and are composed of the following subgroups: (1) Canadian-born descendants of Black people who came from Africa during the slave trade; (2) descendants of Black loyalists, refugees, fugitives and settlers who immigrated during the American civil war, and (3) those who immigrated from the Caribbean and Africa to Canada after World War 2.
Anglophone Afro Caribbean [15]	“Anglophone Afro-Caribbeans were defined as those born in the Commonwealth Caribbean or descendants of recent immigrants from the Commonwealth Caribbean, whose first language was English, and whose lineage was primarily African”.
African Nova Scotians [16]	“We defined African Nova Scotians as anyone who self-identified as “black” in the 2001 census. We based our analysis on an area unit called “community,” as defined by Nova Scotia Community Counts,8 to which census data were recalibrated. In 2001, there were 19 670 African Nova Scotians”.
Caribbean Blacks Non-Caribbean Blacks [17]	Caribbean Blacks are defined as first generation individuals of African descent who migrated from Caribbean countries (see countries listed in footnote to Table 1) … Non-Caribbean Blacks are defined as individuals of African descent who did not migrate from Caribbean countries.
Black, African and Caribbean [18]	Black: Black are most like African Americans with slavery, either in Canada or the United States, part of their heritage. Windsor was a terminus of the Underground Railroad followed by Africans escaping slavery in the United States, and many of their descendants remain in this city and region.” African: In the past 20 years, Windsor has also attracted a sizable population immigrating to Canada from Africa. The majority of this population are immigrants or first-generation Canadians and tend to identify as African, or by their ethnic group (e.g., Ibo, Kikuyu, Hausa, Somali) or heritage country, often maintaining ties to extended family and communities in their heritage countries”. Caribbean: Finally, Windsor is also home to those of African ancestry who arrived in Canada via the Caribbean islands. They too are typically descendants of the slave-trade. Their cultural heritage reflects the historical experiences of slavery combined with their Caribbean background and their origins in Africa. They identify as Caribbean, or by their country of origin (e.g., Jamaica, Dominican Republic). Thus, in Windsor, and in Canada overall, three identities and unique histories comprise the population whose heritage is Africa: Black, African, and Caribbean”.
West Indian [19]	“We use the term “West Indian” to refer to people from the islands and territories in the Commonwealth Caribbean (including Guyana)”.
African, Caribbean, and Black [20, 21]	“Participants were identified as African if they or their parents immigrated from a country in Africa to North America; Caribbean if they or their parents immigrated from a Caribbean country to North America; and Black if they and their parents were born in North America”. “Briefly, African Canadian refers to those who have recently migrated from continental Africa in last few decades. Caribbean-Canadians have also recently immigrated to Canada and can tie their recent familial history to nations in the Caribbean, having arrived at those countries though the trans-Atlantic slave trade. Lastly, Black-Canadians have resided in Canada for hundreds of years”.
Canada’s African Community [22]	Throughout research, the terms African community and African descendants are used inter-changeably to refer to the Black African diasporic population segment that identifies with direct ancestral ties to the continent… Thus, to honor these cultural nuances, the African community in this study is identified as a subset of African immigrants, first-generation African Canadians, and multigeneration African Canadians. When applicable, the phrase continental Africans or terms with similar distinctions will reference Black Africans living within the continent.
African [23]	Participants, or parents of participants, were asked to stipulate the country from which their maternal and paternal ancestors originated. Maternal and paternal ancestry was then classified as European, Middle Eastern, African, Asian, Oceanic, Caribbean, Central and South American, or Aboriginal according to the groupings delineated. *African;* Algeria, Angola, Benin, Botswana, Burkina Faso, Burundi, Cameroon, Cape Verde, Central African Republic, Chad, Democratic Republic of the Congo (Kinshasa, formerly Zaire), Djibouti, Egypt, Equatorial Guinea, Eritrea, Ethiopia, Gabon, Gambia, Ghana, Guinea Bissau, Guinea, Ivory Coast, Kenya, Lesotho, Liberia, Libya, Madagascar, Malawi, Mali, Mauritania, Mauritius, Morocco, Mozambique, Namibia, Niger, Nigeria, Republic of the Congo (Brazzaville), Reunion, Rwanda, Senegal, Seychelles, Sierra Leone, Sao Tome and Principe, Somalia, South Africa, Sudan, Swaziland, Tanzania, Togo, Tunisia, Uganda, Western Sahara, Zambia, Zanzibar, Zimbabwe.
Black/Skin [24] pigmentation	Race/color (ethnicity) was dichotomized to “white” and “other” as proxies for lighter and darker skin pigmentation. The “other” category included black (African, Haitian, Jamaican), American aboriginal, Asian (e.g., Chinese, Vietnamese, Japanese), Latin American, Arab, or Middle Eastern (e.g., Armenian, Turkish, Egyptian).

The term “Black Canadians,” was used by Grégoire-Pelchat and colleagues [95] who explained the term by categorizing Black people into three historical groups. The first category consists of those referred to as “Canadian-born,” described as descendants of Black individuals brought from Africa during the era of slavery. The second category includes Black Loyalists, refugees, fugitives, and settlers who arrived in Canada after the American Civil War. The third category comprises individuals who migrated to Canada from the Caribbean islands and Africa following World War II.

Maticka-Tyndale E et al. [18] utilized different historical contexts to define the term “Black, African, and Caribbean,” providing a detailed explanation for each component. For the term Black, they referred to individuals who escaped slavery in America, as well as those brought directly to Canada as part of the transatlantic slave trade. The term African was defined as individuals who migrated to Canada more recently, specifically within the past twenty years, originating from various African countries. Lastly, the term Caribbean was described as encompassing a history tied to slavery and African origins.

Kerr J et al. [20] and Fante-Coleman T [21] used terminologies similar to the one described earlier, but instead of starting with Black, they began with African, using the terms “African, Caribbean, and Black.” They defined Africans as individuals who migrated from Africa to North America, though they did not specify the timeframe. The Caribbean was defined as individuals with parents who originated from Caribbean islands and later migrated to North America. Blacks were described as individuals whose parents were born in America. The researchers [20, 21] summarized their definitions as follows:

- “African Canadians” are those who migrated to Canada a few decades ago.
- “Caribbean Canadians” are those with ties to the Caribbean, some of whom arrived during the transatlantic slave trade.
- “Black Canadians” are long-standing residents of Canada.

Other researchers [19] provided a definition of West Indian. They explained that this term was used to refer to individuals or populations originating from the islands and territories of the Commonwealth in the Caribbean, including Guyana.

Whitley R., and Kirmayer LJ [15]., also use the term Anglophone Afro-Caribbean, which they define as individuals born in the Commonwealth Caribbean or descendants of recent immigrants from the Commonwealth Caribbean who speak English and have African roots.

Different researchers explain the terminology related to “African” in various ways. According to Davidson and his colleagues, an African is simply someone from Africa, and they provided examples from different countries [23]. In another study by Osazuwa and his colleagues [22], the term “African” was specified to include Canadians and a community descriptor. In this context, it referred to a Black person living in Canada with ancestors from Africa. To distinguish these individuals from African people living in Africa, they used the term “Black of the African continent.”

The last definition by Greene-Finestone et al., [24] highlights that the selection of terminologies related to participants’ race is based on skin pigmentation. Therefore, all individuals with dark skin are referred to as Black.

### Self identification

Aspects of the self-identification process remain ambiguous. While different approaches were employed, some researchers simply stated that ethnicity or race was determined based on self-identification without providing further details on how the process was conducted [96]. Example of this would be in the study by [96]:

“47% (187/400) defined themselves as being of African ethnicity” [96]. In other instances, participants were presented with a set of options to choose from and asked to select one. However, researchers later regrouped these selections into different categories based on their own interpretation, convenience, the source of the data, or definitions provided by a specific organization [27], :

“Race/ethnic background was self-reported and derived from two questions… we categorized self-reported race/ethnic background as: Black, East Asian (Chinese, Japanese, Korean), South or Southeast Asian (SSEA) or White, based on the Canadian Longitudinal Study of Aging (CLSA) nomenclature”.

Some researchers drew existing racial categories from various sources, and then participants were asked to self-identify within these categories. Some sources include the national census, Immigration, Refugees and Citizenship Canada, Statistics Canada, the World Health Organization, population health surveys, district boards, the Canadian Community Health Survey (CCHS), other academic studies, historical contexts, and cardiovascular health research in ambulatory care:

“We defined African Nova Scotians as anyone who self-identified as a Black in the 2001 census” [16].

“Based on categories from Census Canada 2006… resulting in 5 ethnic categories…Black…” [97];

“The ethnically stratified sample was first drawn from census maps” [98].

“We used the Immigration, Refugees and Citizenship Canada Database to identify immigrants to Ontario” [99].

“According to the guidelines of Statistics Canada, patients were categorized into subsets of …. Black…ethnicity” [80].

Some studies involved the administration of surveys or questionnaires where participants were likely provided with a list of racial categories to select from, or in some cases, no predefined options at all. Subsequently, researchers grouped racial categories by relying on their own intuitive understanding [100]. In some cases, researchers used interviews or other qualitative or quantitative methods. They had already defined the population and selected the appropriate terminologies, often relying on communities referred to as Black or those they knew met the inclusion criteria, which helped them identify the participants. Records or observational studies data were also used, and race/ethnicities were drawn.

“Retrospective clinical and sociodemographic data were available for 2,932 patients who fit into one of the four groups: Italian (n = 2,671); white migrant (n = 149); non-white and non-black migrant (n = 71); and black (n = 41). Note that all of the Black patients were migrants from Nigeria, Cameroon, Senegal, Cape Verde or Burundi” [54].

“Reported by physicians on the medical record, ethnicity was defined as ethnic race of the mother and was categorized under: Caucasian, African (including African American)” [54].

## Discussion

The main purpose of this scoping review was to identify the terminologies used to refer to Black populations in Canadian health research and to examine how these populations were defined. Many broad terms used are “Black”, “African”, “Caribbean”, “African Canadian”, “African, Caribbean and Black” (ACB), “Black Canadian”, “African Nova Scotian” and country-specific labels, among others. Some broader umbrella labels such as “BIPOC” or historically terms such as “Negro” were also used. Overall, the findings show considerable variation in how Black populations are named, categorized, and defined across the literature, with some studies using key terms without definition. This lack of clarity complicates interpretation, comparison across studies, and the application of evidence to policy, practice, and community-based interventions, while also limiting alignment with standardized terminology guidance from organizations such as CIHI [7]. 

The characteristics of the included studies show that the literature was largely composed of qualitative, cross-sectional, observational, or other non-intervention designs. This suggests that Canadian health research on Black populations has largely focused on describing experiences, health needs, access to care, and patterns of inequity, rather than testing interventions. The studies concentrated on areas such as clinical health, community health, mental health, and wellness, showing that questions of terminology are relevant across multiple domains of health research. The higher concentration of studies conducted in Ontario may reflect the province’s larger Black population [1] and greater concentration of universities [101]. At the same time, provincial specific terms, such as those linked to African Nova Scotian communities, show why terminology must be sensitive to local histories, identities, and contexts. As one of Canada’s oldest multigenerational Black communities, African Nova Scotians illustrate how naming practices are connected to history, place, and community identity [102]. 

A critical examination of the results shows that the terminologies used to categorize the Black population originate from a flawed foundation rooted in colonial ideologies of race [103]. Colonizers historically erased Africans’ diverse ethnic and cultural identities, imposing homogenizing racial classifications such as “Negro” and subsequently “Black” [104]. This homogenization ignored and supressed the inherent diversity among African people, reducing them to a singular subordinate identity. Compared to “White”, a term symbolizing superiority, goodness, and light, the term “Black” carried negative connotations of “inferiority,” “uncivilized,” and “savage,” with colonial narratives justifying their enslavement and exploitation (hierarchical structure of race) [104]. Consequently, many emergences of diverse rebels show the Blacks’s attempt to redefine themselves and reclaim their lost respect, identities and values [104]. Additionally, the trajectory of Black enslavement [105] is linked to various labels that indicate the regions or areas to which individuals were sent, such as “Caribbean/West Indian,” “Black or African America/Canada,” “Black/African Nova Scotia,” “Black Indigenous,” “Black settlers’, and others. This distinction may reflect a deeper intention and underscores the importance of remembering the histories and injustices that have shaped contemporary identities.

Despite this backdrop, undefined terminologies and inconsistencies in their use in health research may inaccurately represent the population studied and cause unintended harm. These inconsistencies in health research in Canada are problematic for different reasons:

### Ambiguity and misrepresentation

Black, in “Black, African and Caribbean” as defined by Fante-Coleman et al. [18], refers to Blacks who arrived during slavery. The similar term by Kisely et al. [18] (though they go with this order; African, Caribbean, and Black), Black means all Black born in Canada regardless of period. Aside from that, the term ACB itself is devious because there are Africans who are not Black from South Africa and North Africa and Caribbeans who are not Black. The term “Black/African Canadians” can be confusing, especially in studies that do not clearly define these terms. It seems to exclude non-citizen Black individuals, making it difficult to understand.

Another type of ambiguity arises from inconsistencies in terminology, where a single study may use multiple different terms. An example that illustrates this issue is a study entitled “Cancer Care Experiences and the Use of Complementary and Alternative Medicine at End of Life in Nova Scotia’s Black Communities. " [43] In this study, the authors change their terminology throughout the paper. The title refers to “African Canadians in Nova Scotia” but the abstract and aims sections shift to using “Black communities”. In the background section, terms such as “African Canadian,” “Canadian-born Black people,” and “Black settlers in Nova Scotia” are used. Similarly, in the methods section, while the target population is described as African Canadian, the authors introduce additional terms such as “descendants of African Canadians,” “immigrants from the Caribbean and Africa,” “African Nova Scotians,” and “Black communities.” Furthermore, in the data analysis section, the term “people with Black culture” was introduced. Finally, in the discussion section, both “African Canadians” and “Black communities” reappear. Such terminological inconsistencies create confusion and undermine clarity. Inconsistent definitions can hinder accurate interpretation of findings and reduce the coherence of academic discourse. To ensure precision and effective communication, it is imperative that terminologies are used consistently throughout a study, aligning with best practices for academic writing.

Self-identification, often employed as a method for defining the population and categorizing participants, may introduce ambiguity as well. Without clear reporting of methods, it becomes challenging to discern whether it is the participants’ self-identification or the researcher-driven interpretation of identification at play, raising issues of authenticity and potential bias. A relevant example is the study entitled “A Theory-Driven Exploration of Black Canadians’ Psychological Help-Seeking Intentions. " [26] In the methods section, the authors specify that participants’ self-identified racial and ethnic backgrounds included categories such as Caribbean/West Indian, Black, African, African Canadian, African American, multi-ethnic, and other. The authors also note the participant’s places of birth, which include Canada, Jamaica, other Caribbean countries, West Africa, East Africa, the United Kingdom, the United States, and other locations. In the recruitment procedure, the authors specify that participants were recruited through Black community organizations and that eligibility criteria included adults who self-identified as Black or of Caribbean or African descent. This raises questions about whether individuals identified themselves or whether the researchers prompted this identification. The lack of clarity in distinguishing between participant-driven self-identification and researcher-imposed categorizations underscores the need for transparent and detailed reporting of methods to ensure authenticity and mitigate potential biases in population definitions.

### Reliance on assumptions and the possibility of selection and systemic bias

Researchers may rely on societal or personal assumptions that do not reflect the lived experiences or identities of the studied communities. This issue is evident in articles that use “Black” without a clear definition. It can also be seen when studies include a sample of just one individual [106] or fewer than five participants [53, 107] who are presented as representative of the entire Black population.

The descriptors “individuals,” “population,” and “people” are sometimes used without sufficient caution and may introduce bias. For instance, “individuals” may emphasize on singular persons and their uniqueness. The term avoids generalization and humanization but may ignore the systemic issues. “People” focuses on a group of persons but lacks specificity or cultural depth. “Population” identifies groups based on shared characteristics for statistical purposes but risk of dehumanization and overgeneralization. Meanwhile, “communities” can focus on social, cultural and geographic ties, highlighting shared identity and support, but potentially obscuring intra-group diversity.

For example, in a population-based study of chronic Hepatitis C (HCV) in immigrants and non-immigrants in Quebec, Canada [108], the authors concluded that immigrants from intermediate- and high-HCV-prevalence countries are at increased risk for HCV. However, the study has several limitations that may not capture the real world heterogeneity among the population they studied: (a) The study did not differentiate between countries within the Black population; instead grouping them broadly under sub-Saharan Africa region – a region of 49 countries where HCV prevalence vary significantly, ranging from less than 1% to significantly higher rates [109]. (b) Quebec is likely receiving more immigrants from Francophone countries, potentially influencing prevalence data disproportionately. (c) The study compared 1,922 immigrants to 18,940 non-immigrants over 10 years (from 1998 to 2008), with only 157 immigrants representing the 49 sub-Saharan countries. The conclusion that sub-Saharan immigrants have a high prevalence of HCV could be an overgeneralized conclusion. Such aggregation may inadvertently perpetuate misconceptions or discriminatory attitudes.

Similarly, using a broad term like “ACB” to refer to a small group from one country can distort the overall picture of the issue and introduce biases that are specific to certain groups rather than applicable to the entire Black population. An example of this would be in this study where the researchers refer to ACB communities while they recruited only nine individuals predominantly from Nigeria who do not have any affiliation to the Caribbean islands: “Information was derived from a focus group discussion in Windsor with nine faith-based leaders Africans, and primarily Nigerians, head most of the religious congregations that cater to ACB communities.“ [110] Such usage of identifier terms risks conflating distinct identities and misrepresenting the diversity within Black populations.

### Lack of community involvement

As revealed by this scoping review, community involvement in defining relevant terminologies remains limited in most studies. This lack of engagement raises questions about the validity and appropriateness of the terminology used from the community’s perspective. As a result, terms may be used that do not reflect how people identify themselves, fail to respect cultural and historical identities, or lack of accurate representation, lack of relevance, which could cause harm [111]. These risks inappropriate representation, reinforcing inequities, and diminishing community trust toward healthcare systems and research processes.

### Potential for intentional discrimination

The absence of clear definitions and consistent terminology when referring to Black populations can lead to issues of representation that impact cultural and social significance which perpetuate biases or discrimination. Studies that inappropriately presents racial and ethnic terms suggest unequal treatment of identities. For example, in Stratosa et al.‘s study [40], the author wrote: *“*Ethnicity was classified as Asian, black, Caucasian, and other.” Similarly, in another study, the authors wrote: “Overall, our results suggest that East and Southeast Asian, Chinese, South Asian, and black.” These studies that capitalize some racial or ethnic terms while leaving others lowercase for ‘black’ suggest unequal treatment of identities or cultural incompetency of the researchers. This inconsistent approach may reflect unconscious biases or a lack of attention to the cultural importance of terminology. It risks diminishing the significance of Black identities by treating “black” as a mere descriptor rather than acknowledging its broader historical and communal dimensions. Such practices not only create confusion but may also appear dismissive of Black populations’ lived experiences and identities. These examples, which are not isolated incidents but are seen across numerous studies, suggest a systemic disregard for the cultural and social importance of these terms’ cultural and social importance.

Furthermore, this issue is compounded by the tendency to identify other groups based on their country or region of origin (e.g., East Asian or South Asian, Philippine), while referring to “black” solely in terms of skin color—as if Black people do not have places of origin. This practice perpetuates the erasure of Black histories and identities on a global scale and reinforces inequities in how racial and ethnic groups are represented in research [112]. 

### Confusion between ethnicity and race

The classification of race in Canadian health research seems to be confusing, as it is deeply rooted in the historical and social constructs of colonization. Race is a social construct based on physical characteristics such as skin colour, while shared cultural, linguistic, and historical traits define ethnicity [103]. Unfortunately, the concepts of race and ethnicity often gets conflated in Canadian health research. For example, the study “Cardiovascular Risk Factors in Ethnic Populations Within Canada: Results from National Cross-Sectional Surveys” [63] used a data set where the participants were asked to identify their ethnic group from the following categories: “White, Chinese, Japanese, Korean, South Asian, Filipino or Southeast Asian, Black, Latin, Arab, West Asian, Aboriginal, and ‘other [63]. Participants were then grouped into three categories: “White,” “visible minorities” (which included all ethnic categories other than White), and “Aboriginal.” This example highlights the inconsistency in how racial categories, such as Black and White, are treated alongside ethnic groupings like Southeast Asian and country-specific groups, including Japanese and Chinese. The term “Visible Minorities” combines diverse cultural and ethnic groups, which may exhibit different cardiovascular risks. This classification system resembles guidance provided by the Canadian Institute of Health Information (CIHI) [7], classifying White and Black individuals as racial categories. Immigrants from the Asian continent is sorted into subregions such as South, Southeast, and East Asia. This approach adds to the ambiguity of race and ethnicity in healthcare research in Canada [103]. 

### Implications for practice, policy, and future research

The findings of this study highlight the need for standardized terminology in Canadian health research to describe Black populations in Canada accurately. The inconsistent and undefined use of terms to describe research population forces audiences to interpret their meaning, often leading to misrepresentation, inappropriate policies and reinforcing stereotypes. Since health researchers often derive terms from various government and external sources, leading to inconsistencies, national-level data governance is needed to establish standardized terminology for Black communities. This governance can also ensure data protection and prevent unintended misuse. In practice, awareness programs should be implemented to teach researchers about the rich tapestry of Black communities, each with distinct cultures, histories, experiences, immigration statuses, and languages. In the future, researchers need to engage meaningfully with communities and provide clear, thoughtful definitions that respect the identities they aim to describe. Certain specific terms, such as “time in Canada,” “immigration status,” “province/city/location,” “selected communities,” “newcomers,” and “selected individuals,” can be used to replace the broad term in study titles or objectives, mainly when the research involves only a small number of participants but claims to represent the entire Black population. Encouraging self-identification can be beneficial, but improvements are needed to accurately manage the vast ocean of data and prevent researchers from assigning groupings or interpretations based on personal intuition or biases.

### Strengths and weaknesses

Comprehensive search and screening were undertaken across a wide array of academic databases, the gray literature, and through discussions with key informants who deeply understand the subject matter. This extensive process led to a detailed mapping of the various terminologies, labels, and definitions used by the Black population. The engagement of researchers who identify as Black and have a profound connection to the culture and its dynamics played a crucial role in ensuring that the information was interpreted accurately and placed in the appropriate cultural context. Focusing our search specifically on Canada allowed us to delve into the unique characteristics of Black communities within the country. This approach was essential due to the distinct historical narratives and socio-political factors that shape the experiences of Black individuals in the Canadian context. Although we did not conduct a formal quality assessment of the included studies, our findings include a thorough summary of the reported results, organized into relevant subcategories to enhance clarity and understanding. While some time elapsed between the original search and manuscript revision this does not substantially affect the central contribution of the review, as the study was a scoping review rather than a meta-analysis and aimed to map broader patterns in how Black populations are named and defined, rather than to estimate effect sizes or produce pooled findings.

Another limitation of this review is that, while it mapped the terminologies and definitions used to identify Black populations in Canadian health research, it did not conduct a full intersectional analysis of how these terms varied by social determinants such as gender, age, education, socioeconomic status, migration history, refugee status, language, or social capital. Future research should examine how social determinants shape the definition and representation of Black populations, and compare how qualitative, quantitative, and mixed-methods studies use and define related terminology.

## Conclusion

Definition of “Black” in Canadian health research is a complex endeavor that requires careful consideration of historical, cultural, and social contexts. The term “Black” has often been used as an umbrella category, oversimplifying the diverse identities, experiences, and heritage of people of African descent. This lack of nuance risks perpetuating stereotypes and inaccuracies that can undermine the validity and equity of health research. The findings highlight significant inconsistencies and lack of standardization in the terminology used to define and describe Black populations in research. Clear definitions must be revised to ensure the accuracy, relevance, and comparability of study results, potentially leading to misrepresentation and a failure to address these communities’ unique experiences and needs. Lack of community involvement is an important aspect to address. The results underscore the urgent need for standardized guidelines and culturally informed frameworks in health research to ensure inclusivity, precision, and equity in studying Black populations.

## Supplementary Information

Supplementary Material 1.

## Data Availability

All studies included and important extracted data are provided within the manuscript and Additional file 1. The published protocol of this scoping review is available at https://doi.org/10.1136/bmjopen-2023-081296.

## Abbreviations

BIPOC: Black, Indigenous, and People of Colour
ACB: African Caribbean and Black
SSEA: South or Southeast Asian
CLSA: Canadian Longitudinal Study of Aging
HCV: Hepatitis C Virus
CIHI: Canadian Institute of Health Information
RTA: Reflective Thematic Analysis
